# Study on the association between adverse drug reactions to opioids and gene polymorphisms: a case-case–control study

**DOI:** 10.1186/s40360-023-00708-4

**Published:** 2023-11-21

**Authors:** Jing Yang, Ying-zi Sun, Qun-fang Li, Zheng Fu, Yu-yao Guan, Chao Song, Lei Zheng

**Affiliations:** 1 School of Medicine and Pharmacy, Ocean University of China, Qingdao, China; 2 Department of Pharmacy, Shandong Medical College, Jinan, China; 3 Department of Pharmacy, Shandong Provincial Third Hospital, Cheeloo College of Medicine, Shandong University, Jinan, China

**Keywords:** Opioids, Gene polymorphism, Adverse drug reactions (ADRs), Individualized medication, Cancer pain

## Abstract

**Objective:**

Adverse drug reactions (ADRs) caused by opioid drugs show individual differences. Our objective was to explore the association between gene polymorphism and ADRs induced by opioid drugs.

**Methods:**

Evidence-based medical data analysis was conducted for genes related to ADRs induced by opioid drugs to select target genes. Sixty patients with cancer pain who had ADRs after taking opioid drugs (morphine, codeine, oxycodone) and 60 patients without ADRs after taking opioid drugs were used as the experimental group and control group, respectively. Then, we used polymerase chain reaction (PCR) or in situ hybridization to detect target genes. By combining with clinical data such as age, sex, dosage and duration of medication, the effect of gene polymorphism on the ADR of patients after taking opioid drugs was statistically analysed.

**Results:**

Based on a database search and evidence-based medical data, we identified CYP2D6*10, CYP3A5*3, ABCB1, and *OPRM1* as target genes for detection. The results of statistical analysis showed no significant difference in genotype distribution between the experimental group and the control group (*p* > 0.05). However, if 32 patients with ADRs after taking oxycodone and 32 controls were selected for comparison, the SPSS22.0 and SNPStats genetic models showed that the *ABCB1* (062rs1045642) CT and TT genotypes correlated with the occurrence of ADRs (*p* < 0.05): the total number of CT + TT genotypes in the experimental group was 29 (90.62%), with 11 (34.37%) CT + TT genotypes types in the control group.

**Conclusion:**

Polymorphism of *ABCB1* (062rs1045642) is related to ADRs caused by oxycodone, and the incidence of ADRs is higher with the allele T. Polymorphism of *ABCB1* is expected to become a clinical predictor of ADRs to oxycodone, and attention should be given to the occurrence of serious ADRs in patients with *ABCB1* (062rs1045642) CT and TT genotypes.

Opioid analgesics are currently the main treatment for moderate to severe pain. However, individual differences in reactions to these drugs are large, often including nausea, vomiting, lethargy, constipation and addiction, and even respiratory inhibition (RD) and other ADRs. In addition to some cases of drug abuse, the occurrence of the above ADRs is more related to the individual heterogeneity of patients, such as gene polymorphism [1]. At present, it is believed that the genes related to opioid pharmacokinetics and pharmacodynamics include μ opioid receptor (*OPRM1*) (A118G), catechol-O-methyltransferase (*COMT*), *CYP2D6*, CYP3A4*1G, CYP3A5*3, ATP-binding cassette transporter (*ABCB1*); other gene polymorphisms may also be related to the effective analgesic dose of opioids, drug abuse and ADRs. However, the current main problem is that although there are preliminary studies on the correlation between gene polymorphisms and ADRs, there is a lack of high-quality and large-standard clinical research; research to date is mainly on postoperative analgesia patients, and there are some contradictory conclusions [2]. Therefore, the main objective of our study was to determine the correlation between gene polymorphisms (SNPs) and the ADRs caused by opioid drugs used by cancer pain patients and to explain differences in ADRs to opioid drugs among individuals from a genetic perspective, which is helpful to guide individualized clinical drug use [3].

## Methods

### Design and setting

The study was conducted at a 1400-bed tertiary university teaching hospital. This study was approved by the institutional review board of our hospital and complied with the Helsinki Declaration. Each patient provided written consent for study participation before enrolment. The patients were hospitalized from January to December 2022. Sixty patients with cancer pain who had ADRs after taking opioid drugs (morphine, codeine, oxycodone) and 60 patients without ADRs after taking opioid drugs were used as the experimental group and control group, respectively.

### Screening candidate test genes

We searched the release guidelines of the Dutch Pharmacology Working Group (DPWG) and Clinical Pharmacology Implementation Consortium (CPIC), pharmacogenetics (PharmaGKB, FDA, NCCN and OncoKB), PubMed, and Chinese literature (CNKI, Wanfang, China Biomedical Literature Database (CBM)) databases to collect gene loci that may be related to reported adverse reactions of opioid drugs. Then, we preliminarily summarized and analysed the relevant gene loci, determined the recommended level (ABCD level) with reference to CPIC, and carried out gene detection for the target population.

### Screening study population

The Adverse Drug Reaction Monitoring System (CHPS) and the electronic medical record database were used to retrieve the target genes of patients who had adverse drug reactions after use of opioid analgesics in a Class III hospital. The inclusion criteria were as follows: (1) cancer pain patients who experienced adverse drug reactions after using opioid drugs for pain relief; (2) patients with clear awareness and no mental or intellectual disabilities; and (3) patients who voluntarily signed an informed consent form. The exclusion criteria were as follows: (1) liver or kidney dysfunction; (2) long-term use of nonsteroidal antipyretic and analgesic drugs or corticosteroids; (3) history of drug abuse; (4) history of opioid allergy; and (5) history of mental illness or neurological disorder. This study was approved by the Ethics Committee of Shandong Provincial Third Hospital, and all patients signed the informed consent form.

### ADR evaluation criteria

The evaluation criteria for the causal relationship of ADRs adopted the WHO-UMC evaluation method, as follows: ① whether there is a reasonable time relationship between the time sequence of medication and the occurrence of ADRs (ADR occurs after medication); ② whether it is known and whether the suspected ADR conforms to the ADR type known for the drug; ③ excluding others, whether the suspected ADR can be explained by the patient’s pathological state, combined medication, and the effect of therapy; ④ whether the suspected ADR decreases or disappears after the drug is stopped or the dose is reduced; and ⑤ whether the same reaction occurs again after contact with the suspected drug again (recurrence of ADRs can confirm the causal relationship). According to the above criteria, the evaluation results were divided into “affirmative (①-⑤), very likely (①-④), possible, possibly unrelated, to be evaluated, and unable to be evaluated”. Those who were evaluated as “affirmative and very likely” were included in the study.

### Genotype detection

After gargling, the patient used a disposable sampling swab to scrape the mucosa of the sidewall of the oral cavity and collected the exfoliated cells as the monitoring sample (noninvasive, more acceptable to the patient than blood sampling). Samples that were not immediately tested were stored at 2–8 °C for no more than 7 days. The specific detection method was as follows. First, 400 μl of L-sample extraction solution (CQ-ENH type, mainly composed of 0.74% ammonium chloride solution) was added to an oral pharyngeal swab tube, shaken and mixed well, allowed to stand for 1 minute, and then transferred to a 1.5 mL centrifuge tube. After L treatment, 1.0 μl of the sample solution was added to the reagent tube wall; the tube was centrifuged at 12000 r/min for 5 minutes and used for testing. Single-nucleotide polymorphism analysis was performed using PCR or fluorescence in situ hybridization. The PCR method was used to detect *CYP2D6*, and all other genotypes were detected using the in situ hybridization method. The in situ hybridization method was performed using a multichannel fluorescence quantitative analyser Fascan 48S (Shaanxi XZZ No. 20182400043) and a universal genetic testing reagent (Jinan Guangyin Medical Technology Co., Ltd.). Two independent Z-type probes were hybridized with the target sequence in series. The signal amplification precursor sequence binds to 28 bases in the upstream region of the double Z-type probe, causing a change in the three-dimensional configuration of the sequence, thereby amplifying the signal and ultimately outputting genotype detection results.

### Statistical methods

SPSS 22.0 software was used for statistical analysis of data, and the Shapiro Wilk method was used for normality testing of measurement data. Measurement data that conformed to a normal distribution are represented by x ± s, and two independent sample t tests were used for intergroup comparisons. Count data are expressed in terms of examples and percentages or rates, and comparisons between groups were performed using χ2 inspection. The Hardy-Weinberg balance test and correlation analysis between gene polymorphisms and opioid-induced constipation were conducted using the SNPStats program (https://www.snpstats. net/snpstats/start. htm). The correlation analysis results were corrected using the Kolmogorov–Smirnov method. In correlation analysis, statistically significant gene loci and general data such as age, sex, medication dosage, and medication duration were used as variables. Multivariate logistic regression analysis was used to predict the occurrence of opioid-induced constipation. Receiver operating characteristic (ROC) curves were drawn to analyse the effectiveness of each factor in predicting opioid-induced constipation. Inspection level α = 0.05.

## Results

### Identified genes to be tested

Based on a comprehensive database search and evidence-based medical data, 4 target gene loci with strong correlations were identified, including *CYP2D6* (008rs1065852), CYP3A5*3 (058rs776746), *ABCB1* (062rs1045642), and *OPRM1* (047rs1799971). Briefly, we describe the relevant conclusions of the study on the relationship between the above gene loci and adverse drug reactions to opioids and preliminarily determine the degree and grade of correlation with reference to the evidence of PharmGKB. See Table 1 for details.

**Table 1:** Screening of candidate detection genotypes/SNPs

Genotype/SNP	Relationship with ADRs of opioid drugs	PharmGKB evidence level
*CYP2D6* (008rs1065852)	The toxicity of codeine is easily increased in the ultrafast metabolic type. Polymorphism at this site may be related to the gastrointestinal system damage, nervous system damage, respiratory system damage and other toxic reactions caused by opioid drugs.	1A
CYP3A5*3 (058rs776746)	The toxicity of opiates is easily increased in the ultrafast metabolic type. It may be related to the gastrointestinal system damage, nervous system damage, respiratory system damage and other toxic reactions caused by opioid drugs.	2A
*ABCB1* (062rs1045642)	The *ABCB1* gene encodes the P protein, and opioid drugs are the substrate of P protein; polymorphism is related to the respiratory depression caused by opioid drugs and may also be related to increases in other system damage.	3
*OPRM1* (047rs1799971)	Some studies report that polymorphism at this site is related to the gastrointestinal system damage, respiratory system damage and other toxic reactions caused by opioid drugs.	3

### General information of patients

The study population was patients admitted to a tertiary hospital from January to December 2022. They were all cancer patients with pain who used opioid drugs (including oxycodone, morphine and codeine) for analgesia. Patients with adverse ADRs (specifically including 38 cases of constipation, 18 cases of nausea and vomiting, and 8 cases of chest tightness and suffocation) after use of drugs were used as the experimental group, and patients without ADRs were randomly selected as the control group, with 60 cases in each group for gene detection. There was no significant difference in age, height, body mass, daily dosage of drugs or duration of medication between the two groups (*P* > 0.05). See Table 2 for details.

**Table 2:** Comparison of general patient data

Group	n	Male/Female	Age (x¯ ± s), year	Height (x¯ ± s), cm	Weight (x¯ ± s), cm	Daily dose (Calculated by morphine), mg	Duration of medication, day
Experience group	60	32/28	64.21 ± 8.12	166.45 ± 7.80	67.33 ± 11.23	102.05 ± 10.25	99.25 ± 5.20
Control group	60	30/30	62.18 ± 9.36	168.23 ± 8.01	65.45 ± 10.02	100.10 ± 10.50	85.30 ± 8.65

*p* > 0.05

### Distribution of genotype frequency and allele frequency

The allele detection of four genes, *CYP2D6* (008rs1065852), CYP3A5* 3 (058rs776746), *ABCB1* (062rs1045642), and *OPRM1* (047rs1799971), is shown in Table 3 and was consistent with Hardy-Weinberg equilibrium (*P* > 0.05). However, statistical analysis and comparison of genotype distribution between the experimental group and the control group showed no significant difference (*P* > 0.05).

**Table 3:** Genotype distribution of the study population

Group		*CYP2D6* (008rs1065852,C > T)			CYP3A5*3 (058rs776746,A > G)			*ABCB1* (062rs1045642,C > T)			*OPRM1* (047rs1799971,A > G)		
		CC	CT	TT	AA	AG	GG	CC	CT	TT	AA	AG	GG
Experience group	n	20	17	23	36	20	4	24	26	10	28	25	7
	%	33.33%	28.33%	38.33%	60.00%	33.33%	6.67%	40.00%	43.33%	16.67%	46.67%	41.67%	11.67%
Control group	n	15	26	19	35	18	7	31	25	4	30	22	8
	%	25.00%	43.33%	31.67%	58.33%	30.00%	11.67%	51.67%	41.67%	6.67%	50.00%	36.67%	13.33%
χ^2^			0.168			0.121			0.913			0.092	
p			> 0.05			> 0.05			> 0.05			> 0.05	

### Correlation between genotype and ADRs caused by oxycodone

To further evaluate correlations for different drugs, a total of 32 patients with oxycodone (sustained-release tablets or sustained-release capsules) were statistically analysed separately from all 60 patients. First, SPSS 22.0 was used for differential analysis. The results showed that for *ABCB1* (062rs1045642, C > T), significantly more patients with adverse drug reactions carried the CT and TT genotypes than CC genotype (*P* < 0.05, Table 4).

**Table 4:** Genotype distribution of patients using oxycodone

***ABCB1*(062rs1045642,C > T)**									CYP3A5*3(058rs776746,A > G)								
Group		CC	CT	TT	p	C	T	p	Group		AA	AG	GG	p	A	G	p
Experience group	n	3	20	9	0.020	26	38	0.018	Experience group	n	15	16	1	0.330	46	18	0.451
	%	9.38%	62.50%	28.13%		40.63%	59.38%			%	46.88%	50.00%	3.13%		71.88%	28.12%	
Control group	n	21	9	2		51	13		Control group	n	18	11	3		47	17	
	%	65.63%	28.13%	6.25%		79.69%	20.31%			%	56.25%	34.38%	9.38%		73.44%	26.56%	
CYP2D6*10(008rs1065852,C > T)									*OPPM1*(047rs1799971,A > G)								
Group		CC	CT	TT	p	C	T	p	Group		AA	AG	GG	p	G	A	p
Experience group	n	8	12	12	0.700	28	36	0.018	Experience group	n	12	16	4	0.710	40	24	0.770
	%	25.00%	37.50%	37.50%		43.75%	56.25%			%	37.50%	50.00%	12.50%		62.50%	37.50%	
Control group	n	10	13	9		51	13		Control group	n	16	11	5		43	21	
	%	31.25%	40.63%	28.13%		79.69%	20.31%			%	50.00%	34.38%	15.63%		67.19%	32.81%	

In addition, SNPStats (https://www.snpstats.net/snpstats/start.htm) was used for genetic model analysis. The correlation analysis results were corrected by the Kolmogorov–Smirnov method. The *P* value of each SNP was multiplied by the number of genetic markers analysed, and the corrected *P* < 0.05 indicated that the correlation between the site and adverse reactions was significant. The results also showed that the CT genotype and TT genotype with the T allele of *ABCB1* (062rs1045642, C > T) correlated with the occurrence of adverse reactions after use of oxycodone. See Table 5 for the specific results.

**Table 5:** SNPStats genetic model analysis

Genotype/SNP		Experience group	Control group	*p*	OR(95%CI)	AIC	BIC
*ABCB1*(062rs1045642,C > T)	CC	3(9.38%)	21(65.63%)		1	135.4	129.8
	CT + TT	29(90.62%)^*^	11(34.37%)	0.018	0.21(0.09–1.15)		

## Discussion

Opioids are the most common analgesics used for moderate to severe pain and are closely related to the quality of life of cancer patients. However, there are large individual differences in clinical application of these drugs, and the reactions to analgesics of different patients vary greatly. In addition to different analgesic effects, adverse reactions of analgesics (such as nausea, vomiting, lethargy, constipation, and respiratory depression) and even susceptibility to addiction have great individual differences. The reasons for this sensitivity difference include both genetic and nongenetic factors. Genetic factors mainly cause individual differences by affecting the pharmacokinetics (metabolic enzymes and transporters, etc.) and pharmacodynamics (receptors and signal transduction pathways) of opiates [4, 5]. Preliminary studies have been conducted on the correlation between the efficacy and ADRs of opioid analgesics and gene polymorphisms, mainly including *OPRM1* (A118G), COMT, CYP3A4*1G, CYP3A5*3, CYP2D6, and *ABCB1*, among others [6–8]. Relevant studies suggest that the ADRs caused by use of opioids may be closely related to gene polymorphisms of patients. Therefore, to further evaluate relevance in the Asian population, we selected CYP2D6, CYP3A5*3, ABCB1 and *OPRM1* as the target genes for population testing. Unfortunately, no differences were found in the above gene loci when the gene polymorphisms of patients who used morphine, codeine and oxycodone were evaluated; however, if the patients with ADRs after use of oxycodone were counted separately, the analysis result showed that carrying the CT genotype and TT genotype with the T allele of *ABCB1* (062rs1045642, C > T) correlated significantly with the occurrence of adverse drug reactions after use of this drug.

*OPRM1* is the main receptor of most opioid drugs currently used and is also the key target for the effects of analgesia as well as tolerance and dependence. Therefore, the *OPRM1* polymorphism is the main factor affecting the efficacy of opioid drugs. There are multiple mutations in the *OPRM1* gene. For example, A118G is a common SNP. Clinical research shows that this mutation significantly affects the clinical efficacy of opioid drugs [9]. That study found that the analgesic effect of opioid drugs in G118 allele carriers was significantly decreased, and adverse reactions such as respiratory inhibition, nausea and vomiting were less common than in A118 allele carriers. Mague et al. found that G118 carriers were more sensitive to pain; it has also been reported that pain is related to adverse events (constipation, delirium, dizziness, nausea, pain, postoperative nausea and vomiting, itching, respiratory insufficiency, lethargy, urinary retention and vomiting) after use of morphine or oxycodone [10, 11]. However, it should be noted that different results have been reported. For example, some studies report that for patients with severe cancer pain who have not previously used opioid drugs, subjects with the 118G allele need a high dose to control pain, but with no difference in the adverse reactions observed (including nausea, vomiting, constipation, dizziness) [12]. No differences were found in our study. *CYP2D6* is one of the most important oxidative metabolic enzymes in the CYP450 family. Common opioid analgesics such as codeine and tramadol exert pharmacological effects through demethylation by *CYP2D6* into active metabolites as precursors. Current research suggests that *CYP2D6* gene mutation is essential for codeine and tramadol, especially for the *CYP2D6* ultrafast metabolic type. It is recommended that codeine or tramadol should not be used to avoid the risk of severe poisoning. However, no ultrafast metabolic type was found in this study, and no difference was detected. Hydroxycodone is mainly metabolized into oxymorphone and noroxycodone in the liver and intestinal wall through *CYP2D6* and *CYP3A4/5*, respectively. In general, it is believed that *CYP2D6* genotype does not affect cancer pain patients treated with oxycodone [13]. Another associated gene, *CYP3A5,* is the strongest oxidase in the CYP3A pathway that affects metabolism of oxycodone, and CYP3A5*3 is a common mutation site. Polymorphism of this gene can affect the plasma distribution of noroxycodone (a metabolite with a weak analgesic effect of oxycodone), which will lead to an increase or decrease in its dose and an increase in the probability of adverse drug reactions. At present, there are few studies on *CYP3A5* compared with *CYP2D6*. Some studies have shown that individuals carrying the CYP3A5*3/*3 genotype are more likely to develop tolerance to oxycodone, but with no mention of adverse drug reactions. It is also generally believed that AA metabolism is fast and easily increases toxicity according to metabolic type [14, 15]. However, no significant differences were found in our study.

The *ABCB1* (ATP binding cassette subfamily B member - 1) gene encodes P-glycoprotein. *ABCB1* gene polymorphisms may have a close relationship with the pain perception of cancer patients, thus affecting use of opioid drugs by patients. Some scholars have shown that *ABCB1* 3435C > T (rs1045642) patients with different β genotypes have differences in levels of endorphins, resulting in differences in the degree of pain. It has also been suggested that respiratory depression caused by postoperative anaesthesia is related to adverse drug reactions. The incidence of respiratory depression after using fentanyl in patients with the 1236TT, 2677TT and 3435TT genotypes is high, with a deep degree of inhibition [14]. Genotype is also related to a decrease in P-gp protein transport function. That study found that expression of P-gp in the duodenum of CC carriers is more than 2 times higher than that of TT carriers and that the *ABCB1* (rs1045642) T/T genotype can lead to a decrease in the drug clearance rate and the possibility of adverse reactions caused by an increase in the blood concentration of the drug [16, 17]. Therefore, the CT + TT genotype was present at a significantly higher rate among patients in the oxycodone adverse drug reaction group than the CC genotype, which may be related to the slow metabolism of oxycodone and increase in blood drug concentration.

## Conclusion

Our preliminary study shows that the CT and TT genotypes with the T allele of *ABCB1* (062rs1045642, C > T) are related to the occurrence of adverse reactions after oxycodone use and are expected to become a clinical predictor of adverse reactions to oxycodone drugs. Overall, the occurrence of severe constipation should be given more attention in patients who have been taking drugs for a long time. However, the limitation of this study is that the number of patients included was small, which cannot greatly reflect the overall impact of population polymorphism, and more cases will be included in future work. In general, cancer patients with pain are greatly affected by tumour type and stage, leading to sensitivity and heterogeneity issues. Further research will include the same tumour type and stage to eliminate this impact.

## Data Availability

The datasets generated and/or analysed during the current study are available in the Annotare 2.0 repository, [https://www.ebi.ac.uk/fg/annotare/edit/17619/].
